# Socioemotional wellbeing of mixed race/ethnicity children in the UK and US: Patterns and mechanisms

**DOI:** 10.1016/j.ssmph.2018.06.010

**Published:** 2018-06-22

**Authors:** James Nazroo, Afshin Zilanawala, Meichu Chen, Laia Bécares, Pamela Davis-Kean, James S. Jackson, Yvonne Kelly, Lidia Panico, Amanda Sacker

**Affiliations:** a Cathie Marsh Institute, University of Manchester, Humanities Bridgeford Street, Oxford Road, Manchester M13 9PL, UK; b University College London, London, UK; c University of Michigan, Ann Arbor, MI, USA; d Institut National d’Etudes Demographiques, Paris, France

**Keywords:** UK, United Kingdom, US, United States, MCS, Millennium Cohort Study, ECLS-B, Early Childhood Longitudinal Study-Birth Cohort, Mixed race, Mixed ethnicity, Wellbeing, Childhood, Inequality

## Abstract

Existing literature suggests that mixed race/ethnicity children are more likely to experience poor socioemotional wellbeing in both the US and the UK, although the evidence is stronger in the US. It is suggested that this inequality may be a consequence of struggles with identity formation, more limited connections with racial/ethnic/cultural heritage, and increased risk of exposure to racism.

Using data from the UK Millennium Cohort Study (n = 13,734) and the US Early Childhood Longitudinal Study-Birth Cohort (n ~ 6250), we examine differences in the socioemotional wellbeing of mixed and non-mixed 5/6 year old children in the UK and US and explore heterogeneity in outcomes across different mixed groups in both locations. We estimate a series of linear regressions to examine the contribution of factors that may explain any observed differences, including socio-economic and cultural factors, and examine the extent to which these processes vary across the two nations.

We find no evidence of greater risk for poor socioemotional wellbeing for mixed race/ethnicity children in both national contexts. We find that mixed race/ethnicity children experience socio-economic advantage compared to their non-mixed minority counterparts and that socio-economic advantage is protective for socioemotional wellbeing. Cultural factors do not contribute to differences in socioemotional wellbeing across mixed and non-mixed groups.

Our evidence suggests then that at age 5/6 there is no evidence of poorer socioemotional wellbeing for mixed race/ethnicity children in either the UK or the US. The contrast between our findings and some previous literature, which reports that mixed race/ethnicity children have poorer socioemotional wellbeing, may reflect changes in the meaning of mixed identities across periods and/or the developmental stage of the children we studied.

## Highlights

• Mixed race/ethnicity children are thought to have poorer socioemotional wellbeing
• We find no evidence that mixed race/ethnicity children have poorer socioemotional wellbeing in a study covering children aged 5/6 in the US and UK
• We find that mixed race/ethnicity children do have socio-economic advantage
• This socio-economic advantage is protective for socioemotional wellbeing

## Introduction

A striking change in developed countries is the rapidly increasing numbers of mixed race/ethnicity people (McCubbin et al., 2013, Rees et al., 2011). Existing literature suggests that mixed race/ethnicity children are more likely to experience emotional, psychological and behavioral (socioemotional) difficulties than their non-mixed minority counterparts. This increased risk is considered to be independent of demographic and economic factors (Udry, Li, & Hendrickson-Smith, 2003) and to be a consequence of struggles with identity formation and more limited connections with the cultural heritage of parents (Bratter & Eschbach, 2005; Cooney & Radina, 2000; Lorenzo‐Blanco, Bares, & Delva, 2013; Root, 1992; Schlabach, 2013; Tizard & Phoenix, 2002; Udry et al., 2003), alongside increased risks of exposure to both overt racism (Alibhai-Brown, 2001, Ifekwunigwe, 2001) and more subtle forms of discrimination (Nadal, Sriken, Davidoff, Wong, & McLean, 2013). Mixed race/ethnicity children may face the experience of being caught between two socially significant categories, being denied one, or the other, or both, so being thought of as having a less than ‘authentic’ racial/ethnic identity and, consequently, ‘cultural homelessness’ (Vivero & Jenkins, 1999). Bhui (2002) points to the negative psychological consequences of such challenges to identity and how this might have consequences for educational outcomes, employment and health in adulthood (Duncan & Brooks-Gunn, 1997). In addition, the multi-race/ethnic family itself may be stigmatized.

Nevertheless, some have argued that mixed race/ethnicity people suffer less racial discrimination than their non-mixed minority counterparts, because they may appear more white (Twine & Gallagher, 2008), perhaps because they have more flexibility in their management of a racialised identity. In addition, there is some evidence that mixed race/ethnicity children have more favorable socio-economic circumstances than their non-mixed minority peers (Bratter & Kimbro, 2013; Panico & Nazroo, 2011). Both more favorable socio-economic circumstances and reduced exposure to racism and racial discrimination are likely to result in improved socioemotional wellbeing (Kelly, Becares, & Nazroo, 2013; Priest et al., 2013).

Indeed, there are some exceptions to the findings on the potential socioemotional difficulties faced by mixed race/ethnicity children (Danko et al., 1997, Platt, 2012). Here it is worth noting that concerns have been expressed about the generalizability of existing findings. As Cooney and Radina (2000) have noted, much of the research, although not all (Bratter & Kimbro, 2013), has been limited to clinical settings, generating the presentation of troubled mixed race/ethnicity children who struggle with identity formation and who have socioemotional problems in their families, schools, and communities. This is aggravated by the failure of some studies to include comparisons between mixed race/ethnicity children and their non-mixed counterparts.

When considering the generalizability of findings, it is also important to consider the socially constructed nature of race and ethnic categories. This means that the identified associations between race/ethnic categories and outcomes are a consequence of how these categories are constructed, understood and acted upon. However, the social and personal significance of race/ethnic categories will vary across periods, contexts and nations, meaning cohorts that grow up in different circumstances will potentially have different experiences. Importantly, most research on mixed race/ethnicity comes from the US, so it is possible that prior findings are specific to that context. For example, in the US levels of segregation are particularly marked in demographic, social and economic terms. It was only in 1967 that the Supreme Court ruled that anti-miscegenation laws were unconstitutional, reflecting the ongoing significance of the ‘one drop’ rule (a historical, but still prominent, social and legal framework whereby someone with any African ancestry, however distant, is considered to be Black). Such a context might lead to mixed identities being experienced as particularly problematic in the US, and indeed not identified as such, something that might be present to a lesser extent in other nations such as the UK, perhaps because the presence of large numbers of non-white people is a relatively new phenomenon in the UK, following migration from Commonwealth countries in the 1950s and 1960s. Indeed, patterns of settlement and migration are very different in the US and UK, with the potential for marked differences in the processes of identifying and attributing meaning to race/ethnic categories. This provides a very different context for ‘mixing’ across socially significant race/ethnic boundaries in the two countries, making it important to extend research in this field beyond the US. In addition, the socially constructed meaning of ethnic/race identities makes it important to examine the heterogeneity of circumstances and outcomes across different types of mixed identity. Finally, much of the research on this topic has been conducted during adolescence, a particularly vulnerable developmental period, so there is limited understanding of how poor socioemotional wellbeing might develop earlier in childhood.

This paper uses data from the UK Millennium Cohort Study (MCS) and the US Early Childhood Longitudinal Study-Birth Cohort (ECLS-B) to examine differences in the socioemotional wellbeing of mixed and non-mixed children during early childhood in the UK and US, and the factors that might underlie any differences. We have two core hypotheses: that mixed race/ethnicity children will experience poorer socioemotional wellbeing than their non-mixed minority counterparts, as well as their White counterparts; and that the risk for this will be greater in the US than in the UK. In addition, we explore heterogeneity across mixed race/ethnicity groups, and hypothesized mechanisms related to socio-economic position and cultural identity.

## Methods

### Data source

We use data from the MCS and the ECLS-B, which are comparable birth cohort studies that follow children from infancy. Both are nationally representative and contain relevant information on children and their families.

The MCS sampled children born between 2000 and 2002, who were identified through Child Benefit records (Plewis, Calderwood, Hawkes, Hughes, & Joshi, 2007). The sample is clustered at the electoral ward level (an administrative unit), with oversampling of ethnic minority populations, disadvantaged residential areas, and the three smaller UK countries (Northern Ireland, Scotland, and Wales). The ECLS-B sampled children who were born in 2001, using birth certificate data from the National Center for Health Statistics vital statistics registry (Nord et al., 2004). Twin and low and very low birth weight babies were oversampled, in addition to American Indian, Chinese, and Other Asian/Pacific Islander children.

This study uses data collected from the MCS children at the age 5 wave and collected at the kindergarten wave of the ECLS-B children (age 5–6), which involved a random subsample of about 85% of the children (Snow et al., 2007). All analyses were weighted to adjust for nonresponse and included sample design factors. All sample sizes reported from the ECLS-B data are rounded to the nearest 50 in accordance with Institute of Education Sciences (IES) reporting rules, which are designed to minimize the risk of disclosure.

Our analytic sample includes singleton and twin births in both datasets. We excluded children who were reported to have attention-deficit/hyperactivity disorder, autism, or Asperger’s syndrome. The sample was exclusive to children for whom a caregiver’s report of socioemotional wellbeing was available and for whom race/ethnicity was reported. Two further sample exclusions were made for the ECLS-B: the very small number of children who had missing observations for more than two-thirds of the items comprising externalizing and internalizing behavior; and, following IES rules on small cell sizes, those who had missing data on family structure, equivalized household income, housing tenure, or maternal employment. The analytic sample was 13,734 in the MCS and approximately 6250 in the ECLS-B.

### Measures

In MCS, children’s socioemotional wellbeing was assessed with the Strengths and Difficulties Questionnaire (SDQ) (Goodman, 1997), completed by the main caregiver (usually the mother). This is a 25-item instrument asking questions about five domains of social and emotional wellbeing: conduct problems, hyperactivity, emotional symptoms, peer problems, and prosocial behavior. Consistent with existing practice, which is based on both theoretical propositions and analysis of the measurement properties of items included in the SDQ measure (Goodman, Lamping, & Ploubidis, 2010), scores from the conduct problems and hyperactivity domains were summed to construct an externalizing behavior score, and scores from the emotional symptoms and peer problems domains were summed to construct an internalizing behavior score. Each score was analyzed as a continuous variable with higher scores indicating a tendency toward poorer behavior.

In the ECLS-B, mothers reported on their children’s socioemotional wellbeing. An externalizing behavior score was constructed by taking the mean of seven items that asked about children’s temper tantrums, aggressive, annoying, destructive, angry, impulsive, and overly active behaviors. An internalizing behavior score was constructed from two items asking if a child ‘worried about things’ or ‘seemed unhappy’. Each item was rated on a 5-point Likert scale. Higher scores reflect a tendency towards poorer behavior. Although the externalizing and internalizing score items are not drawn from a single behavioral scale, they are consistent with previous comparative evidence using similar parent-reported behavior items (Washbrook, Waldfogel, Bradbury, Corak, & Ghanghro, 2012).

Race and ethnic categories were constructed using the respondent’s report of the child’s race/ethnicity and, where available, reports of the mother’s and father’s race/ethnicity. Within each study data collection was based on census categories, so, while the classification approach varied across the studies, it reflected national demography.

In the MCS, race/ethnicity was categorized as mixed if the main respondent chose a mixed category or if the race/ethnic categories for the child’s parents were reported as different (Panico & Nazroo, 2011). If the main respondent chose a ‘mixed’ ethnic category for the child, but the categories of the child’s natural parents were the same, we reclassified the child according to the parents’ ethnicity. Race/ethnic categories used for analysis were: White; Indian; Pakistani; Bangladeshi; Black Caribbean; Black African; and the ‘mixed’ counterpart for each of these, including White mixed children with parents from more than one White ethnic group (for example, White British and White North American, or White British and White Polish).

In ECLS-B, we also used the full categorization of the child and his/her parents’ race and ethnicity, but included birth certificate data where self-reports of race and ethnicity were missing. We then aggregated the full list of categories to a smaller number that were: Non-Hispanic White, Black (including both Non-Hispanic and Hispanic), Black-White (including both Non-Hispanic and Hispanic), Mexican Hispanic, Mexican Hispanic-White, Non-Mexican Hispanic, Non-Mexican Hispanic-White, Non-Hispanic American Indian and Non-Hispanic American Indian-White. From this list of categories we consider the following categories as mixed: Black-White, Mexican Hispanic-White, Non-Mexican Hispanic-White, and Non-Hispanic American Indian-White. Here we move beyond simply studying mixed race to also incorporate mixed ethnicity, making the conceptual approach compatible with that used for the UK. For simplicity, hereafter we use the abbreviation NH in place of Non-Hispanic.

As described in the introduction, to explore possible heterogeneity in outcomes in both the UK and US we retain the full range of mixed categories in the analysis, rather than combine them.

We investigate the importance of three groups of covariates to mixed race/ethnicity differences in children’s socioemotional wellbeing: child, demographic and parenting context; cultural factors; and socio-economic circumstances (see the appendix for the construction of these variables and their association with children’s socioemotional wellbeing).

Child characteristics included were age, gender, and an indicator for twin birth. Demographic factors were mother’s age at time of birth and single parenthood. Parenting context covered maternal depression, assessed with the six-item version of the Kessler questionnaire (Kessler et al., 2002) in the MCS and 12 items from the Center for Epidemiologic Studies Depression Scale (CES-D) (Radloff, 1977) in the ECLS-B, and discipline strategies, using a composite score of seven items taken from the Conflict Tactics Scale (Straus & Hamby, 1997) in the MCS, and 6 comparable items covering discipline strategies for a misbehaving child in the ECLS-B.

Socio-economic factors included equivalized family income, highest parental occupation class, highest parental educational attainment, housing tenure and maternal employment.

Cultural factors were assessed using binary indicators of English as the primary language spoken at home, whether the mother was foreign-born, and whether a grandparent lives in the household. Although not direct indicators of cultural identity and cultural practice, we considered each of these to indirectly reflect closeness to cultural traditions.

The small proportion of missing data for the independent variables is presented in Table 1, Table 2 and the relationship between missing values and outcome variables is shown in Table A1, Table A2 in the appendix. Despite this low-level of non-response at the item level, we re-ran our analyses of the MCS data (which had the largest proportion of missing data) using Multiple Imputation by Chained Equations to explore the impact of missing data on the original model estimations. We imputed 20 datasets and consolidated results from all imputations using Rubin’s (2004) combination rules. The comparison between this model and the model we include in the paper revealed no meaningful difference; coefficients were identical or near identical and statistical significance was consistent. The imputed model is available from the authors on request.

**Table 1:** Children’s socioemotional wellbeing and explanatory factors by mixed race/ethnicity: UK (Mean or %).

	White non-mixed	White mixed	Indian non-mixed	Indian mixed	Pakistani non-mixed	Pakistani mixed	Bangladeshi non-mixed	Bangladeshi mixed	Black Caribbean non-mixed	Black Caribbean mixed	Black African non-mixed	Black African mixed
	n=11,405	n=444	n=299	n=52	n=447	n=44	n=143	n=10	n=142	n=174	n=222	n=59
**Socioemotional wellbeing**												
Externalizing behavior [0–20]	4.7	4.0*^,^^a^	4.5	3.9	5.8*	4.4^a^	5.2	3.8	5.9*	5.3*	4.2	5.0
Internalizing behavior [0–18]	2.3	2.0*^,^^a^	2.9*	2.2^a^	4.1*	3.4*	3.6*	2.5	3.0*	2.7	2.7	2.8
												
**Child characteristics**												
Child age (years)	5.2	5.2	5.2	5.2	5.2	5.2	5.2	5.3	5.2	5.2	5.2	5.2
Male child	50.4	55.2	50.5	65.9	47.0	54.8	48.5	54.8	55.7	47.0	49.5	41.3
Twin birth	1.4	1.4	0.2	1.6	0.5	3.2	0.0	0.0	1.3	3.1	3.4	0.0
												
**Demographic factors**												
												
Mother’s age at time of birth, years												
14–19	8.0	1.5	1.5	2.8	5.6	11.4	7.2	14.9	10.2	9.4	2.1	1.3
20–24	15.7	5.7	20.8	12.5	35.0	17.2	38.5	21.8	16.9	23.9	10.0	25.0
25–29	28.4	22.7	37.4	28.8	33.2	33.4	39.8	48.1	25.2	19.7	21.2	23.4
30–34	31.1	41.4	27.2	34.8	19.4	21.7	13.1	15.2	18.9	28.7	31.6	36.7
35–51	16.8	28.8	13.2	21.2	6.8	16.3	1.4	0.0	28.8	18.3	35.1	13.7
Missing	1.6	0.5	4.3	1.9	2.5	0.0	2.1	0.0	1.4	2.9	7.2	1.7
Single parent	17.9	10.2	6.2	17.7	13.4	19.9	6.3	14.9	61.1	36.3	42.0	40.4
												
**Parental context**												
												
Maternal depression												
No risk for depressive symptoms	97.1	99.0	94.1	99.3	92.3	95.1	92.7	100.0	94.9	98.0	93.0	95.6
Risk for depressive symptoms	2.9	1.0	5.9	0.7	7.7	4.9	7.3	0.0	5.1	2.0	7.0	4.4
Missing	0.9	0.5	8.0	1.9	20.8	9.1	22.4	10.0	7.7	2.3	24.3	5.1
												
Discipline strategies score												
Normal	69.3	71.6	82.6	74.9	69.3	90.5	80.1	86.4	67.6	62.7	78.3	79.7
High	30.7	28.4	17.4	25.1	30.7	9.5	19.9	13.6	32.4	37.3	21.7	20.3
Missing	1.3	1.1	9.7	1.9	22.4	11.4	25.9	10.0	7.7	2.9	27.0	5.1
												
**Cultural factors**												
												
Language spoken at home												
English only or mostly	99.3	92.4	58.2	93.8	40.6	79.5	22.2	43.0	100.0	99.2	69.2	85.0
English or other; Other only	0.7	7.6	41.8	6.2	59.4	20.5	77.8	57.0	0.0	0.8	30.8	15.0
												
Mother’s migration												
UK born	96.9	61.3	46.8	75.8	44.1	60.0	10.6	49.5	80.5	93.7	25.1	70.2
Foreign born	3.1	38.7	53.2	24.2	55.9	40.0	89.4	50.5	19.5	6.3	74.9	29.8
Missing	8.7	8.1	9.7	13.5	12.8	13.6	17.5	10.0	16.9	13.8	23.4	11.9
Resident grandparent	2.3	1.5	30.3	6.1	15.4	15.1	13.7	11.2	5.4	1.7	2.3	0.5
												
**Socio-economic factors**												
												
Equivalized household income												
Lowest quintile	16.4	10.2	14.3	11.8	47.7	45.6	54.9	23.9	46.9	32.0	37.7	38.0
Second quintile	18.9	15.7	25.3	16.9	35.1	26.7	27.4	4.7	21.8	22.6	22.9	15.0
Third quintile	21.4	14.6	19.5	10.0	10.0	12.5	9.5	12.3	13.4	18.6	16.8	18.4
Fourth quintile	21.9	20.3	23.1	19.1	2.4	5.4	4.2	52.1	9.8	14.8	10.4	10.3
Highest quintile	21.3	39.3	17.8	42.2	4.8	9.8	4.0	6.9	8.0	12.0	12.2	18.3
Missing	0.1	0.0	0.0	0.0	0.4	0.0	0.0	0.0	0.7	0.6	0.5	0.0
												
Highest parental occupation												
Not working	17.1	9.4	11.3	14.6	38.6	28.1	38.5	14.9	41.5	29.3	41.8	45.0
Semi-routine/routine	13.6	4.4	15.5	11.3	16.4	11.2	22.6	17.1	13.2	11.8	10.1	8.2
Supervisory/technical	6.4	4.1	4.8	1.6	5.4	1.0	8.8	4.7	3.9	3.8	2.8	1.3
Small employer/self-employed	8.9	10.5	12.5	7.9	21.3	24.4	8.8	0.0	1.4	5.4	2.8	3.5
Intermediate	10.9	7.2	15.2	0.0	5.0	4.8	1.9	0.0	9.7	10.0	8.5	6.0
Managerial/professional	43.1	64.5	40.8	64.6	13.2	30.4	19.4	63.3	30.3	39.8	33.9	36.0
Missing	0.1	0.5	0.3	0.0	0.0	0.0	0.0	0.0	0.0	0.0	0.5	0.0
												
Highest parental educational attainment												
None	5.3	1.7	6.8	2.7	18.5	13.6	16.2	0.0	10.7	4.3	15.4	15.4
Overseas	1.1	0.8	3.6	1.5	10.0	1.6	9.1	9.0	1.4	2.2	7.3	6.8
NVQ1	5.3	1.0	3.3	0.0	7.3	6.4	8.1	0.0	4.6	7.8	1.9	0.9
NVQ2	25.0	15.2	13.9	9.2	22.9	17.7	27.3	14.9	26.2	30.7	14.4	14.5
NVQ3	16.6	11.5	12.2	9.3	13.4	16.2	14.3	12.8	13.2	14.8	5.8	17.5
NVQ4	37.5	45.6	39.2	40.4	20.4	29.6	19.4	11.0	37.4	33.1	37.8	30.7
NVQ5	9.2	24.2	21.1	36.9	7.5	15.0	5.6	52.3	6.5	7.1	17.5	14.1
Missing	0.0	0.2	0.0	0.0	0.2	0.0	0.0	0.0	0.0	0.0	0.5	0.0
												
Housing tenure												
Own	67.6	80.3	80.9	81.9	72.1	47.0	53.5	52.1	29.4	43.0	30.6	23.7
Private rent	8.6	7.5	2.1	12.1	6.5	15.5	5.9	14.6	7.4	11.3	5.2	21.7
Public rent	21.5	11.0	6.6	5.9	14.5	26.0	35.6	22.1	60.6	44.0	63.4	54.6
Other	2.3	1.1	10.4	0.0	6.9	11.5	5.0	11.2	2.7	1.7	0.9	0.0
Missing	0.0	0.0	0.0	0.0	0.2	0.0	0.0	0.0	0.0	0.0	0.0	0.0
												
Maternal employment												
Not working	38.6	37.2	34.9	34.9	84.5	64.7	78.0	84.8	50.6	42.5	52.7	53.7
Working	61.4	62.8	65.1	65.1	15.5	35.3	22.0	15.2	49.4	57.5	47.3	46.3

Note: All means are weighted by MCS3 sample weights. Sample sizes are unweighted. The number of observations differs by variable and range is 12,434–13,734. This is exclusive to singleton and twin birth and respondents who are biological, step, adopted, or foster mothers. Children who had ADHD/Asperger’s or Autism were excluded. Figures on missing data for each variable are on a sample with observed ethnicity and an interview at MCS3.

* p<0.05 (two-tailed tests); Significant differences are in reference to White non-mixed children and only evaluated for socioemotional wellbeing.

a Significant differences at p<0.05 (two-tailed tests) within ethnicity and between mixed and non-mixed counterpart.

**Table 2:** Children’s socioemotional wellbeing and explanatory factors by mixed race/ethnicity: US (Mean or %).

	NH White	Black	Black-White	Mexican Hispanic	Mexican Hispanic-White	Non-Mexican Hispanic	Non-Mexican Hispanic White	NH American Indian	NH American Indian-White
	n~2400	n~950	n~150	n~200	n~500	n~100	n~250	n~100	n~300
**Socioemotional wellbeing**									
Externalizing behavior [1–5]	2.2	2.3*	2.3	2.3	2.3	2.2	2.3	2.4*	2.3
Internalizing behavior [1–5]	2.2	1.9*	1.9*	2.1	2.2	2.1	2.2	2.0*	2.2
									
**Child characteristics**									
Child age (years)	5.4	5.4	5.4	5.4	5.4	5.4	5.4	5.4	5.5
Male child	49.7	51.1	48.2	62.9	48.5	47.6	48.8	55.7	38.3
Twin birth	3.5	2.9	2.0	2.3	2.0	1.7	3.0	+	2.3
									
**Demographic factors**									
									
Mother’s age at time of birth, years									
15–19	7.1	18.6	7.7	15.8	14.7	16.4	9.8	19.5	8.1
20–24	19.2	31.9	35.4	28.5	30.7	36.6	27.0	36.2	44.9
25–29	28.6	22.9	28.5	25.7	26.5	30.1	23.6	18.8	20.2
30–34	29.0	15.6	22.8	15.2	17.0	10.9	27.2	15.8	17.7
35–50	16.2	11.1	5.6	14.9	11.2	+	12.5	9.6	9.0
									
Family structure									
Two parents	89.5	42.3	65.2	84.5	80.4	80.1	72.2	61.5	79.2
Single parent	10.5	57.7	34.8	15.5	19.7	19.9	27.8	38.5	20.8
									
**Parental context**									
									
Maternal depression (12 items)									
None	66.3	53.5	48.0	60.1	60.1	61.9	66.2	52.3	45.5
Mild	20.3	23.4	31.6	22.7	23.8	26.4	15.4	22.7	27.2
Moderate/severe	13.4	23.1	20.4	17.2	16.0	11.8	18.4	25.0	27.3
Valid N	~2350	~950	~100	~200	~500	~100	~250	~100	~250
Missing	2.8	2.9	+	+	5.3	+	5.1	6.3	7.3
									
Discipline strategies (0–6)									
Normal	86.6	85.9	87.8	88.7	86.1	89.8	84.9	91.6	81.9
High	13.4	14.1	12.2	11.3	13.9	10.2	15.1	8.4	18.1
									
**Cultural factors**									
Language spoken at home primarily not English	2.3	5.2	6.8	79.1	58.9	86.8	46.1	4.4	+
Mother is foreign born	4.0	11.5	12.2	72.2	55.5	77.8	48.1	+	+
Resident grandparent	15.0	31.4	22.7	19.7	28.8	33.9	32.1	49.8	29.0
									
**Socio-economic factors**									
									
Equivalized household income									
Lowest quintile	9.5	43.4	25.1	38.0	31.5	41.0	22.3	46.6	21.9
Second quintile	14.0	21.8	19.4	39.6	35.0	23.1	21.7	26.6	23.3
Third quintile	20.9	18.0	25.4	14.0	18.5	17.0	22.3	14.1	21.0
Fourth quintile	27.6	10.4	14.9	5.1	10.1	17.0	16.3	9.8	22.3
Highest quintile	28.1	6.4	15.1	3.2	4.9	+	17.4	+	11.5
									
Highest parental occupational prestige score									
Lowest quintile	16.2	25.5	13.1	41.4	27.7	27.9	23.9	27.1	29.2
Second quintile	19.8	27.9	32.6	44.2	39.1	39.4	28.6	35.4	32.4
Third quintile	16.1	18.1	13.9	6.4	8.5	13.6	13.4	13.6	4.1
Fourth quintile	23.3	14.4	27.7	3.8	15.6	11.3	19.8	10.2	22.1
Highest quintile	24.6	14.1	12.7	4.3	9.2	7.7	14.3	13.7	12.2
Valid N	~2300	~750	~100	~200	~450	~100	~200	~100	~250
Missing	4.1	21.8	15.0	10.0	7.6	+	11.2	19.2	11.5
									
Highest parental educational attainment									
Less than high school	3.4	12.8	4.6	39.0	26.8	28.9	10.3	9.9	6.3
High school/GED	16.3	38.7	35.3	35.1	35.1	38.8	27.2	36.7	37.0
Some college	31.7	34.1	36.6	22.1	26.4	16.5	35.1	45.7	35.0
Bachelor degree or higher	48.5	14.4	23.5	3.8	11.7	15.7	27.4	7.6	21.7
									
Housing tenure									
Own home	76.0	26.0	40.4	39.8	49.2	34.0	48.1	35.9	53.8
Rent house or townhouse	9.9	23.7	23.1	20.8	18.4	23.9	16.2	21.7	16.4
Rent apartment or condominium	5.8	39.1	22.5	28.8	23.6	39.0	24.6	20.8	17.8
Other	8.3	11.1	14.1	10.7	8.8	+	11.1	21.6	12.0
									
Maternal employment									
Not working	36.7	31.1	34.2	50.0	48.7	29.6	41.0	32.3	40.9
Working	63.3	68.9	65.8	50.0	51.3	70.4	59.0	67.7	59.1

Note: All means are weighted by W4R0 and are based on valid cases for each factor. Sample sizes are unweighted and are rounded to the nearest 50 given IES reporting rules. The analytic sample is exclusive to respondents who were biological, step, adopted, or foster mothers of singleton and twin births. Children who had ADHD or Autism or missing data on outcome (socioemotional wellbeing) or race were excluded. Children with missing data on family structure, equivalized household income, housing tenure, or maternal employment were also excluded due to too few cases according to IES rules. The analytic sample is around 6250.

+ Cell size is not available due to IES reporting rules.

No significant differences were found between mixed and non-mixed groups within ethnicity.

* p<0.05 (two-tailed tests); Significant differences are in reference to NH White children and only evaluated for socioemotional wellbeing.

### Analytic Strategy

We estimated a series of regressions to examine the contribution of explanatory factors to differences in externalizing and internalizing behaviors across race/ethnic groups. Taylor-linearized variance estimation was used to obtain standard errors. These regressions were carried out in three steps:

• Model 1 presents estimates of race/ethnic differences controlling only for child characteristics and demographics;
• Model 2 adjusts for child characteristics, and socioeconomic factors;
• Model 3 adjusts for child characteristics and cultural factors;
• Model 4 simultaneously adjusts for child characteristics, demographics, parenting context, socio-economic factors and cultural factors.

Each of these models uses the White non-mixed group in the UK, and NH White group in the US as the primary comparison, allowing for an examination of differences between that group and each of the non-mixed and mixed minority groups, and an examination of whether any observed inequalities are consistent across non-mixed and mixed children within the same broad race/ethnic group. For the descriptive analyses presented in Table 1, Table 2 each non-mixed and equivalent mixed group is also directly compared, and statistically significant differences are footnoted.

## Results

Table 1, Table 2 present differences in explanatory factors by race/ethnicity for the UK and US, respectively. In both countries, mixed children compared with their non-mixed counterparts lived in socio-economically advantaged households. Mixed children in the UK lived in less culturally traditional households when compared with their non-mixed minority counterparts. This pattern was also evident in the US among Mexican Hispanic-White and Non-Mexican Hispanic-White children, and, to a lesser extent among Black-White and NH American Indian-White children, compared with their non-mixed minority counterparts.

Table 3, Table 4 show the association between children’s socioemotional wellbeing and race/ethnicity, controlling for a range of explanatory factors, in the UK and US respectively. For each table, the upper panel shows mean differences in externalizing behaviors, and the lower panel shows mean differences for internalizing behaviors. In the UK, White and Indian mixed children were less likely to have externalizing behaviors, compared with White non-mixed children. This advantage was not present for Indian non-mixed children, and was largely explained by the socio-economic advantage of White and Indian mixed children. Both Pakistani and Black Caribbean non-mixed children were more likely to have externalizing behaviors than White non-mixed children, but this disadvantage was not present for Pakistani mixed children and was smaller for Black Caribbean mixed children. For most groups, controlling for cultural factors made little difference, although it increased the disadvantage for Black Caribbean children relative to White non-mixed children. In the fully adjusted model much of the increased risk for Pakistani and Black Caribbean non-mixed children is explained, and the increased risk for Black Caribbean mixed children is fully explained. Once their socio-economic disadvantage had been accounted for (model 2), non-mixed Black African children were less likely to have externalizing behaviors compared with White non-mixed children, but this advantage was not shared by Black African mixed children.

**Table 3:** Regression estimates predicting externalizing and internalizing behavior: UK (*n* = 13,734).

	Model 1: Child characteristics + demographics	Model 2: Child characteristics + socio-economic	Model 3: Child characteristics + cultural factors	Model 4: Fully adjusted
**Externalizing behavior**				
White mixed	-0.35*	-0.21	-0.67***	-0.095
	(0.17)	(0.17)	(0.17)	(0.16)
				
Indian non-mixed	-0.083	0.026	-0.33	0.17
	(0.20)	(0.20)	(0.24)	(0.19)
				
Indian mixed	-0.81	-0.38	-0.94*	-0.24
	(0.44)	(0.44)	(0.43)	(0.43)
				
Pakistani non-mixed	0.95***	0.68**	1.05***	0.49*
	(0.21)	(0.23)	(0.23)	(0.22)
				
Pakistani mixed	-0.39	-0.63	-0.38	-0.19
	(0.37)	(0.42)	(0.40)	(0.41)
				
Bangladeshi non-mixed	0.26	-0.16	0.39	-0.14
	(0.42)	(0.42)	(0.42)	(0.41)
				
Bangladeshi mixed	-1.09	-0.64	-0.92	-0.37
	(0.87)	(0.69)	(1.05)	(0.51)
				
Black Caribbean non-mixed	0.79**	0.57*	1.17***	0.55*
	(0.24)	(0.28)	(0.23)	(0.26)
				
Black Caribbean mixed	0.48	0.37	0.71**	0.25
	(0.26)	(0.23)	(0.26)	(0.25)
				
Black African non-mixed	-0.46	-1.01***	-0.51*	-0.84***
	(0.24)	(0.23)	(0.25)	(0.23)
				
Black African mixed	0.18	-0.19	0.37	0.099
	(0.54)	(0.51)	(0.55)	(0.51)
				
**Internalizing behavior**				
White mixed	-0.19	-0.13	-0.43***	-0.12
	(0.11)	(0.10)	(0.11)	(0.11)
				
Indian non-mixed	0.60**	0.60**	0.32	0.45*
	(0.21)	(0.21)	(0.23)	(0.20)
				
Indian mixed	-0.14	0.037	-0.23	0.048
	(0.25)	(0.24)	(0.24)	(0.23)
				
Pakistani non-mixed	1.74***	1.38***	1.53***	1.09***
	(0.23)	(0.24)	(0.23)	(0.23)
				
Pakistani mixed	1.03*	0.82*	0.94*	0.85
	(0.41)	(0.40)	(0.41)	(0.44)
				
Bangladeshi non-mixed	1.16***	0.75**	0.87**	0.43
	(0.29)	(0.27)	(0.28)	(0.25)
				
Bangladeshi mixed	0.11	0.11	-0.044	0.017
	(0.62)	(0.59)	(0.76)	(0.62)
				
Black Caribbean non-mixed	0.47	0.33	0.69*	0.29
	(0.33)	(0.31)	(0.32)	(0.30)
				
Black Caribbean mixed	0.22	0.15	0.34	0.14
	(0.21)	(0.21)	(0.21)	(0.21)
				
Black African non-mixed	0.35	0.0021	0.20	-0.16
	(0.21)	(0.18)	(0.24)	(0.18)
				
Black African mixed	0.36	0.11	0.39	0.15
	(0.37)	(0.38)	(0.37)	(0.37)

Standard errors in parentheses

Notes: All models are adjusted for sample design with weights from MCS 3. White non-mixed is the reference group. Child characteristics are age, gender, and twin status. Demographic factors are mother’s age at birth of child and family structure. Socio-economic factors are household equivalized income, highest parental occupational class, highest parental education, housing tenure, and maternal employment. Cultural factors are English spoken at home, foreign born, and resident grandparent. Psychosocial factors are maternal depression and discipline strategies.

*** p<0.001,

** p<0.01,

* p<0.05 (two-tailed tests)

**Table 4:** Regression estimates predicting externalizing and internalizing behavior: US (*n* ~ 6250).

	Model 1: Child characteristics + demographics	Model 2: Child characteristics + socio-economic	Model 3: Child characteristics + cultural factors	Model 4: Fully adjusted
**Externalizing behavior**				
Black	-0.01	-0.04	0.07*	-0.06
	(0.04)	(0.04)	(0.04)	(0.04)
				
Black-White	0.04	0.03	0.10	0.00
	(0.07)	(0.07)	(0.07)	(0.06)
				
Mexican Hispanic	0.07	-0.04	0.08	-0.03
	(0.06)	(0.06)	(0.07)	(0.07)
				
Mexican Hispanic-White	0.05	-0.02	0.07	-0.02
	(0.04)	(0.04)	(0.05)	(0.05)
				
Non-Mexican Hispanic	-0.04	-0.12	-0.02	-0.10
	(0.08)	(0.08)	(0.08)	(0.08)
				
Non-Mexican Hispanic White	0.04	0.01	0.07	0.01
	(0.06)	(0.06)	(0.07)	(0.06)
				
NH American Indian	0.13	0.07	0.17*	0.08
	(0.07)	(0.07)	(0.07)	(0.07)
				
NH American Indian-White	0.11	0.09	0.13	0.05
	(0.15)	(0.14)	(0.14)	(0.14)
				
**Internalizing behavior**				
Black	-0.31***	-0.28***	-0.30***	-0.31***
	(0.04)	(0.04)	(0.04)	(0.04)
				
Black-White	-0.25***	-0.23**	-0.26**	-0.27***
	(0.08)	(0.08)	(0.08)	(0.07)
				
Mexican Hispanic	-0.03	0.00	-0.17*	-0.15*
	(0.06)	(0.07)	(0.07)	(0.07)
				
Mexican Hispanic-White	0.01	0.05	-0.09	-0.08
	(0.05)	(0.05)	(0.05)	(0.05)
				
Non-Mexican Hispanic	-0.08	-0.05	-0.23***	-0.23**
	(0.07)	(0.08)	(0.07)	(0.07)
				
Non-Mexican Hispanic White	0.02	0.04	-0.06	-0.07
	(0.05)	(0.05)	(0.05)	(0.05)
				
NH American Indian	-0.21***	-0.18**	-0.20***	-0.17***
	(0.06)	(0.06)	(0.06)	(0.05)
				
NH American Indian-White	0.01	0.04	0.02	0.01
	(0.11)	(0.10)	(0.11)	(0.11)

Standard errors in parentheses.

Note: All models are adjusted for sample design with w4rps for PSU, w4rst for strata, and w4r0 for weight for ECLS-B 2006 Kindergarten parent-child sample. Non-Hispanic White is the reference group. Child characteristics are age, gender, and twin status. Demographic factors are mother’s age at birth of child and family structure. Socio-economic factors are household equivalized income, highest parental occupational prestige score, highest parental education, housing tenure, and maternal employment. Cultural factors are English spoken at home, bio-mother foreign born, and resident grandparent. Psychosocial factors are maternal depression and discipline strategies.

*** p<0.001,

** p<0.01,

* p<0.05 (two-tailed tests)

White mixed children also had fewer internalizing behaviors compared with their non-mixed White counterparts, and again this was largely explained by their socio-economic advantage. For Indian, Black Caribbean and Bangladeshi children, those in the non-mixed category had a disadvantage in relation to internalizing behaviors when compared with White non-mixed children, but this disadvantage was not shared by their mixed counterparts. In the case of Pakistani children, however, both non-mixed and mixed children had a greater risk of internalizing behaviors, albeit this was to a lesser degree for mixed Pakistani children and their greater risk was no longer statistically significant in the fully adjusted model.

For the US, findings were more straightforward. The only significant differences were for Black and NH American Indian children, who had higher externalizing behavior scores compared with NH White children when cultural factors were included in the model, a finding that was not apparent in other steps of the model, nor in the fully adjusted model. There was no evidence of an increased risk of externalizing behaviors for those in mixed categories. There were few differences also in risk of internalizing behaviors, with a reduced risk for Black, Black-White mixed and NH American Indian compared with NH White children. These differences were not altered in the fully adjusted model. However, in fully adjusted models, Mexican Hispanic and non-Mexican Hispanic children had a reduced risk of internalizing behaviors compared with NH White children.

## Discussion

In this paper we examined the possibility that mixed race/ethnicity children were at risk of poor socioemotional wellbeing in the UK and the US, how this might vary across these two national contexts and across different mixed race/ethnicity groups within these countries, and the factors that might underlie, or mitigate, this greater risk. To do this we used data from two comparable studies, the Millennium Cohort Study (MCS) in the UK and the Early Childhood Longitudinal Study-Birth Cohort (ECLS-B) in the US. This allowed us to study markers of socioemotional wellbeing, in terms of both internalizing and externalizing behaviors, around age 5.

We found no evidence to support our hypothesis, which proposed that in both locations mixed race/ethnicity children would have poorer socioemotional wellbeing. We also found no evidence to support our hypothesis that any greater risk for poorer socioemotional wellbeing among mixed race/ethnicity children would be more apparent in the US than in the UK, because of the more racialized nature of mixed identities in the US. We did, however, find that mixed race/ethnicity children were in a socio-economically advantaged position. This socio-economic advantage related to improved socioemotional wellbeing. We also found that mixed/race ethnicity children had weaker ties to minority cultures; however, we found no evidence to suggest that this was related to differences in socioemotional wellbeing.

These findings are in contrast to most of the existing literature on mixed race/ethnicity children in both the US and the UK (Bratter & Eschbach, 2005; Cooney & Radina, 2000; Lorenzo‐Blanco et al., 2013; Root, 1992; Schlabach, 2013; Tizard & Phoenix, 2002; Udry et al., 2003), although not all (Danko et al., 1997, Platt, 2012). It is possible that the differences between our findings and those that have identified poorer socioemotional wellbeing among mixed race/ethnicity children are a consequence of differences in the age ranges between studies. The children included in our analyses were aged 5 or 6, while most other research has concentrated on adolescence; it may be that adolescence is a developmental stage where children are particularly at risk of having their identities threatened as they attempt to establish independence. This may be even more the case for mixed race/ethnicity children, because of the possibility that the perceived (by themselves and others) heterogeneity of their identities means that they cannot fully identify with their parents or with their peers. Evidence from the UK suggests that for some of the mixed race/ethnicity groups studied here there is an increasing risk of poor socioemotional wellbeing as they move into adolescence, particularly for mixed Pakistani and mixed Bangladeshi children, although this is not the case for all groups (Zilanawala, Sacker, & Kelly, 2016). Consequently, it may be that the analysis we have provided at the age of 5/6, when children enter their early school years, provides a baseline from which adverse outcomes might develop as they become more widely socialized and pass through adolescence. However, it is worth noting that from the age of three children develop an understanding of racial and ethnic cognition, and can identify the racial/ethnic identity of themselves and of others (Quintana, 1999).

It is also important to place our findings and those of others within historical and national context. As argued earlier, race and ethnicity and, consequently, mixed race/ethnicity, are social constructs, so the notion of an identity as mixed, what counts as mixed race/ethnicity, and the meanings that such a label carries, is socially and historically contingent (Aspinall & Song, 2013). This, of course, underpins our purpose in conducting a comparison between the circumstances and outcomes for mixed race/ethnicity children in the UK and the US. Our expectation was that these identities would be more negatively perceived in the US than the UK, because of factors such as the legacy of the ‘one drop’ rule, higher levels of segregation, and differing patterns of migration and settlement. However, our findings did not support this hypothesis.

In terms of historical period, it might also be expected that mixed identities were more likely to be perceived as reflecting transgressive relationships in the past than they are now. Indeed, in the US context, where hypodescent has been an important factor, those with a Black and White heritage may have been previously identified as Black.^1^ The difference between our findings and those from previous studies may reflect such a change in the personal and social meanings of mixed race/ethnicity identities and a growing acceptance and presence of such identities. However, alongside this we recognize ongoing evidence of experiences of racism and discrimination, prejudicial attitudes, and consequent broader social, economic and health inequalities in both the UK and US (Bailey et al., 2017, Wallace et al., 2016).

This study used a detailed mixed race/ethnicity classification to understand the risks for poor socioemotional wellbeing in two nationally representative cohorts. However, it has some limitations. We examined cross-sectional associations, meaning we cannot explore change with age/developmental stage, nor time ordered associations. Future research should more directly investigate developmental patterns and causal mechanisms. Data limitations also meant that we could not explore some potentially important mechanisms, such as differential experiences of racism and composition of social networks.

Nevertheless, this study provides important new evidence. Outside of the US, Latin America and Brazil, relatively little is yet known about the experiences, identity options and socio-economic backgrounds of mixed race/ethnicity people (Song, 2012). Intriguingly, though, recent evidence suggests that mixed race (Black-White) adults in Canada experience poorer mental health than either their White or Black counterparts (Veenstra, 2017). This points to the need to add to the very few studies that attempt to draw comparisons across nations with different historical, social and economic circumstances (Bratter & Eschbach, 2005). In addition, we have been reasonably comprehensive in our coverage of race/ethnic groups in both the US and UK, allowing us to explore the possibility of heterogeneity in outcomes across mixed groups within nations.

In conclusion, this paper makes a significant contribution to our understanding of the relationship between mixed race/ethnicity and children's socioemotional wellbeing. We did not find that mixed race/ethnicity children were at risk of poor socioemotional wellbeing at the age of 5/6, and in many cases they were advantaged, in contradiction to much of the existing literature. Future research should explore causal pathways more directly and how these vary across contexts, cohorts and developmental stage.

## Funding source

The authors were supported by grants from the UK Economic and Social Research Council (ES/J019119/1) and the National Institutes of Health (1RO1HD061294-01). Dr Zilanawala received additional support from a grant from the UK Economic and Social Research Council ES/R003114/1) The funders had no role in the interpretation of these data or in the writing of this article.

## Declarations of interest

None.

## Ethics approval

None.

## Figures and Tables

**Table A1:** Weighted means of socioemotional wellbeing by explanatory factors: UK.

	Externalizing behavior [0–20]	Internalizing behavior [0–18]
**Demographic factors**		
		
Mother’s age at time of birth, years		
14–19	6.0	3.1
20–24	5.7	2.9
25–29	4.7	2.4
30–34	4.2	2.1
35–51	4.0	2.1
*P*-value	<0.001	<0.001
Missing	4.7	2.8
		
Family structure		
2-parent household	4.4	2.3
1-Parent household	5.7	3.0
*P*-value	<0.001	<0.001
		
**Socio-economic factors**		
		
Equivalized household income		
Lowest quintile	5.8	3.2
Second quintile	5.3	2.8
Third quintile	4.6	2.3
Fourth quintile	4.1	2.1
Highest quintile	3.7	1.7
*P*-value	<0.001	<0.001
Missing	6.4	1.5
		
Highest parental occupation		
Not working	5.9	3.3
Semi-routine/routine	5.4	2.8
Supervisory/technical	4.9	2.4
Small employer/self-employed	4.6	2.3
Intermediate	4.6	2.2
Managerial/professional	3.9	2.0
*P*-value	<0.001	<0.001
Missing	4.0	1.7
		
Highest parental educational attainment		
None	6.6	3.7
Overseas	5.8	3.5
NVQ1	5.9	3.1
NVQ2	5.3	2.6
NVQ3	4.7	2.4
NVQ4	4.0	2.1
NVQ5	3.5	1.9
*P*-value	<0.001	<0.001
Missing	5.3	4.9
		
Housing tenure		
Own	4.1	2.1
Private rent	5.3	2.6
Public rent	5.9	3.1
Other	5.3	2.6
*P*-value	<0.001	<0.001
Missing	2.9	3.4
		
Maternal employment		
Not working	5.2	2.9
Working	4.3	2.1
*P*-value	<0.001	<0.001
Missing	10.0	3.0
		
**Cultural factors**		
		
Language spoken at home		
English only or mostly	4.7	2.4
English or other; Other only	5.0	3.4
*P*-value	0.041	<0.001
Missing	3.0	0.0
		
Mother’s migration		
UK born	4.6	2.3
Foreign born	4.6	2.9
*P*-value	0.649	<0.001
Missing	5.2	2.7
		
Resident grandparent		
No	4.7	2.4
Yes	5.2	2.8
*P*-value	0.003	0.006
		
**Parenting context**		
		
Maternal depression		
No risk for depressive symptoms	4.6	2.3
Risk for depressive symptoms	7.3	4.6
*P*-value	<0.001	<0.001
Missing	6.1	4.0
		
Discipline strategies score		
Normal	3.9	2.2
High	6.3	2.8
*P*-value	<0.001	<0.001
Missing	5.9	3.8

Note: All means are weighted by MCS3 sample weights. Sample sizes are unweighted. The sample on which this analysis is based is 13,734. This is exclusive to singleton and twin birth and respondents who are biological, step, adopted, or foster mothers. Children who had ADHD/Asperger’s or Autism were excluded. Significant differences indicate joint significance of an explanatory variable, are two-tailed tests, and exclude the missing categories.

**Table A2:** Weighted means of socioemotional wellbeing by explanatory factors: US.

	Externalizing behavior [1–5]		Internalizing behavior [1–5]
**Demographic factors**			
		
Mother’s age at birth of child, years			
15–19	2.3		2.1
20–24	2.3		2.1
25–29	2.3		2.1
30–34	2.2		2.1
35–50	2.2		2.1
*P*-value	p<.001		0.471
		
Family Structure			
Two parents	2.2		2.1
Single parent	2.4		2.1
*P*-value	p<.001		0.067
		
**Socio-economic factors**			
		
Equivalized household income			
Lowest quintile	2.4		2.1
Second quintile	2.3		2.1
Third quintile	2.2		2.1
Fourth quintile	2.2		2.1
Highest quintile	2.2		2.2
*P*-value	p<.001		0.094
		
Highest parental occupational prestige score (valid n~5700)			
Lowest quintile	2.3		2.1
Second quintile	2.3		2.0
Third quintile	2.3		2.1
Fourth quintile	2.2		2.1
Highest quintile	2.2		2.2
*P*-value	p<.05		p<.001
Missing	2.4		2.2
		
Highest parental educational attainment			
Less than high school	2.4		2.1
High school/GED	2.3		2.0
Some college	2.2		2.0
Bachelor degree or higher	2.2		2.2
*P*-value	p<.001		p<.001
		
Housing tenure			
Own home	2.2		2.1
Rent house or townhouse	2.3		2.1
Rent apartment or condominium	2.3		2.0
Other	2.4		2.1
*P*-value	p<.001		p<.05
		
Maternal employment status			
Mother not working	2.3		2.1
Mother working	2.3		2.1
*P*-value	0.469		0.096
		
**Cultural factors**			
		
Language spoken at home			
Primarily English	2.3		2.1
Primarily not English	2.3		2.2
*P*-value	0.333		p<.001
		
Mother’s migration			
U.S. born	2.3		2.1
Foreign born	2.2		2.2
*P*-value	0.438		p<.001
		
Resident grandparent			
No	2.3		2.1
Yes	2.3		2.1
*P*-value	0.116		0.184
		
**Parenting context**			
		
Maternal depression (12 items; valid n~6000)			
None	2.2		2.0
Mild	2.4		2.2
Moderate/severe	2.5		2.3
*P*-value	p<.001		p<.001
Missing	2.2		2.1
		
Discipline strategies (0–6)			
Normal	2.2		2.1
High	2.5		2.2
*P*-value	p<.001		p<.001

Note: All means are weighted by W4R0. Sample sizes are unweighted and are rounded to the nearest 50 given IES reporting rules and are around 6250 unless indicated otherwise. The analytic sample is exclusive to respondents who were biological, step, adopted, or foster mothers of singleton and twin births. Children who had ADHD or Autism or missing data on outcome (socioemotional wellbeing) or race were excluded. Children who had missing data on family structure, equivalized household income, housing tenure, or maternal employment were also excluded due to too few cases according to IES rules. A significant test has excluded the missing category and indicates the joint significance of an explanatory variable and is a two-tailed test adjusting for complex survey design.

## Appendix A Construction of independent variables and their relationship with wellbeing outcomes

### Independent variables

#### Demographic factors

• Child’s age and gender, and an indicator for twin birth.
• Mother’s age at time of birth (<19, 20–24, 25–29, 30–34, > 34 years).
• Single parenthood.

### Socio-economic factors

• Equivalized family income in quintiles
• Highest parental occupation class. For the UK, occupational class is measured using the National Statistics Socio-economic Classification (NS-SEC) categories: semi-routine and routine, supervisory and technical, small employer and self-employed, intermediate, and managerial and professional, with an additional category of not working. For the US, parental occupation was measured using a prestige score and then converted to quintiles.
• Highest parental educational attainment. The UK education variable consisted of 7 categories on the National Vocational Qualification (NVQ) equivalence scale: NVQ5 Higher degree, NVQ4 First degree/diploma, NVQ3 A/AS levels, NVQ2 GCSE grades A-C, NVQ1 GCSE grades d-G, Overseas qualification, None. NVQ3 is roughly equivalent to achieving a secondary or high school diploma. For the US, the education variable was measured using 4 categories: less than high school, high school diploma or General Educational Diploma (GED), some college, and bachelor’s degree or higher.
• Housing tenure (own, private rent/rent house or townhouse, public rent/rent apartment or condominium, and other)
• Maternal employment.

### Cultural factors

• Whether English was the primary language spoken at home.
• Whether Mother was foreign-born.
• Whether a grandparent lives in the household.

### Parenting context

• Mother’s mental health. In the UK data this was assessed with the six-item version of the Kessler questionnaire (ranging 0–24)(Kessler et al., 2002), with a binary indicator constructed to indicate risk for depressive symptoms (score greater than 12). In the US data this was assessed using 12 items from the Center for Epidemiologic Studies Depression Scale (CES-D) (ranging 0–36)(Radloff, 1977), with a three category variable constructed for mental distress: none (< 5), mild (5–9), and moderate/severe (>9).
• Discipline strategies in the UK data was a composite score of seven items (how often do you do the following when child is ‘naughty’: ignore, smack, shout, send to bedroom/naughty chair, take away treats, tell off, and bribe) taken from the Conflict Tactics Scale (ranging 0–28, α=0.71)(Straus & Hamby, 1997). A binary indicator was constructed to indicate high scores (16–28 or more than 1 standard deviation above the mean (mean=11.2; SD=4)) and normal scores (0–15)(Y Kelly, Sacker, Del Bono, Francesconi, & Marmot, 2011). In the US data, six comparable items covering discipline strategies for a misbehaving child were selected and summed (ranging 0–6): ignore child, use timeouts, give a warning, deny privileges, spanking or hitting, yelling or threatening). Similar to the MCS, a binary indicator was used to distinguish normal scores (0–4) from high scores (5–6). Both measures capture harsh disciplinary practices used by parents.

Each explanatory variable included a ‘missing’ category to capture item non-response. (Table A1, Table A2).

## Footnotes

1 We are grateful to a reviewer of an earlier version of this paper who made this point.
